# Target Low-Density Lipoprotein-Cholesterol and Secondary Prevention for Patients with Acute Myocardial Infarction: A Korean Nationwide Cohort Study

**DOI:** 10.3390/jcm11092650

**Published:** 2022-05-08

**Authors:** Ju Hyeon Kim, Jung-Joon Cha, Subin Lim, Jungseok An, Mi-Na Kim, Soon Jun Hong, Hyung Joon Joo, Jae Hyoung Park, Cheol Woong Yu, Do-Sun Lim, Kyeongmin Byeon, Sang-Wook Kim, Eun-Seok Shin, Kwang Soo Cha, Jei Keon Chae, Youngkeun Ahn, Myung Ho Jeong, Tae Hoon Ahn

**Affiliations:** 1 Department of Cardiology, Cardiovascular Center, Korea University Anam Hospital, Korea University College of Medicine, Seoul 02841, Korea; jhnkim86@gmail.com (J.H.K.); joonletter@hanmail.net (J.-J.C.); subinlim.dasos@gmail.com (S.L.); alsk1229@gmail.com (M.-N.K.); psyche94@hanmail.net (S.J.H.); drjoohj@gmail.com (H.J.J.); jhpark3992@naver.com (J.H.P.); ycw717@naver.com (C.W.Y.); dslmd@kumc.or.kr (D.-S.L.); 2 Department of Pathology, Korea University Anam Hospital, Korea University College of Medicine, Seoul 02841, Korea; anbox@naver.com; 3 Department of Cardiology, Heart and Brain Institute, Chung-Ang University Gwang-Myeong Hospital, Chung-Ang University College of Medicine, Gwangmyeong-si 14353, Korea; kmbyeon79@gmail.com (K.B.); swkimcv@cau.ac.kr (S.-W.K.); 4 Division of Cardiology, Department of Internal Medicine, Ulsan University Hospital, University of Ulsan College of Medicine, Ulsan 44033, Korea; sesim1989@gmail.com; 5 Division of Cardiology, Department of Internal Medicine, Pusan National University Hospital, Busan 49241, Korea; cks@pusan.ac.kr; 6 Division of Cardiology, Department of Internal Medicine, Chonbuk National University Medical School, Jeonju 54907, Korea; jkchae@jbnu.ac.kr; 7 Division of Cardiology, Department of Internal Medicine, Chonnam National University Hospital, Chonnam National University Medical School, Gwangju 61469, Korea; cecilyk@hanmail.net (Y.A.); myungho@chollian.net (M.H.J.)

**Keywords:** myocardial infarction, low-density lipoprotein cholesterol, statin, secondary prevention

## Abstract

Although lowering low-density lipoprotein cholesterol (LDL-C) levels following acute myocardial infarction (MI) is the cornerstone of secondary prevention, the attainment of recommended LDL-C goals remains suboptimal in real-world practice. We sought to investigate recurrent adverse events in post-MI patients. From the Korea Acute Myocardial Infarction-National Institutes of Health registry, a total of 5049 patients with both measurements of plasma LDL-C levels at index admission and at the one-year follow-up visit were identified. Patients who achieved an LDL-C reduction ≥ 50% from the index MI and an LDL-C level ≤ 70 mg/dL at follow-up were classified as target LDL-C achievers. The primary endpoint was a two-year major adverse cardiac and cerebrovascular event (MACCE), including cardiovascular mortality, recurrent MI, and ischemic stroke. Among the 5049 patients, 1114 (22.1%) patients achieved the target LDL-C level. During a median follow-up of 2.1 years, target LDL-C achievers showed a significantly lower incidence (2.2% vs. 3.5%, log-rank *p* = 0.022) and a reduced adjusted hazard of MACCE (0.63; *p* = 0.041). In patients with acute MI, achieving a target LDL-C level was associated with a lower incidence and a reduced hazard of recurrent clinical events. These results highlight the need to improve current practices for managing LDL-C levels in real-world settings.

## 1. Introduction

The latest guideline for the management of dyslipidemia emphasizes a subgroup of individuals at very high risk for recurrent atherosclerotic cardiovascular disease (ASCVD) events [1]. Lowering the low-density lipoprotein cholesterol (LDL-C) level is the cornerstone of the secondary prevention of ASCVD events, as numerous clinical trials have shown a decreased risk of ASCVD-related morbidity and mortality, especially among patients with acute myocardial infarction (MI) [2,3,4]. However, studies of patients with predefined characteristics are often not representative of those seen in daily practice. In addition, the attainment of LDL-C target goals remains suboptimal in real-world settings [5,6], and more evidence is needed to assess the attainment of recommended LDL-C goals among very high-risk patients in the era of popular combination therapies for LDL-C reduction. Thus, here we sought to investigate recurrent ASCVD events in post-MI patients who did or did not achieve LDL-C target goals and evaluate the relationship between LDL-C changes and clinical outcomes.

## 2. Materials and Methods

### 2.1. Source of Data

The Korean Acute Myocardial Infarction Registry-National Institutes of Health (KAMIR-NIH) registry is a prospective, multicenter, observational, web-based cohort database. Patients diagnosed with acute MI at hospital presentation were enrolled at 20 major centers in Korea that were capable of performing primary percutaneous coronary intervention (PCI). The KAMIR-NIH protocols were verified and approved by the institutional review board of each participating center, and written informed consent was provided by each participant upon enrollment. All data were collected by independent clinical research coordinators, using a web-based case report form in the Internet-based Clinical Research and Trial Management System (iCReaT), a data management system established by the Centers for Disease Control and Prevention, Ministry of Health and Welfare, Republic of Korea (iCReaT Study No.C110016; KCT-0000863). The detailed study design and protocol were published previously [7].

### 2.2. Study Population

**Figure 1:**
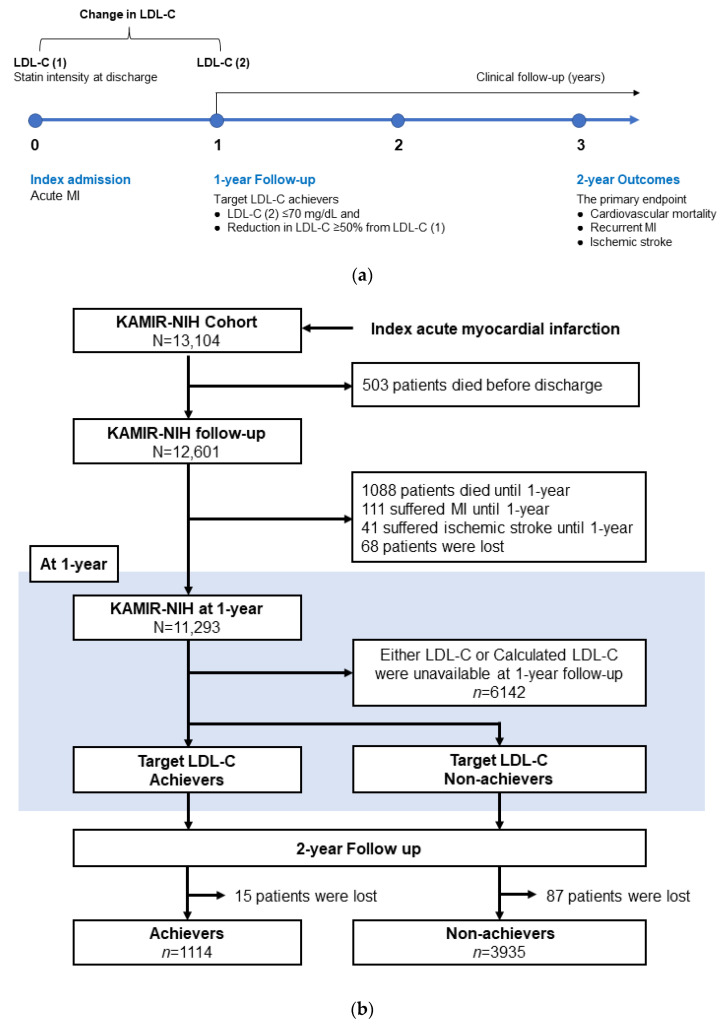
Study population. Flow chart of the study (**a**) and patient demographics according to flow chart (**b**). KAMIR-NIH, Korean Acute Myocardial Infarction Registry-National Institutes of Health; LDL-C, low-density lipoprotein cholesterol; MI, myocardial infarction.

From November 2011 to December 2015, a total of 13,104 patients were enrolled in the KAMIR-NIH registry. Among them, patients who were lost to follow-up, those who died during the index admission (*n* = 503), and those with all-cause mortality (*n* = 1088) at 1-year follow-up were excluded. Patients who experienced any MI (*n* = 111) or ischemic stroke (*n* = 41) until the one-year follow-up visit were also excluded in order to create a more homogeneous risk population. Patients for whom the measurements of plasma LDL-C levels at the index admission and the 1-year follow-up were available were included in the present analysis. The diagnosis of index MI was confirmed on invasive coronary angiogram in all study subjects. Patients who completed a three-year clinical follow-up since the index MI event were divided into target LDL-C achievers and non-achievers, excluding those without any clinical follow-up information (Figure 1). Patients who achieved an LDL-C reduction ≥ 50% from baseline and an LDL-C level ≤ 70 mg/dL (1.8 mmol/L) at the one-year follow-up were classified as target LDL-C achievers.

### 2.3. Study Variables and Endpoints

Chronic kidney disease (CKD) was defined as baseline serum creatinine level > 2.0 mg/dL. Blood samples from baseline laboratory tests were drawn at the time of the index admission, and lipid profiles, including LDL-C, were measured using standard enzymatic methods after an 8-h fast. LDL-C levels were assessed using the Friedewald formula (LDL-C [mg/dL] = total cholesterol − high-density lipoprotein cholesterol − [triglyceride/5]) when direct measurement values were unavailable [8], and patients with a triglyceride level ≥ 400 mg/dL were excluded. High sensitivity C-reactive protein (hs-CRP) levels were analyzed by immunoturbidimetric analysis. The left ventricular ejection fraction (LVEF) was determined by transthoracic echocardiography using the modified Simpson’s biplane method. Statin usage and intensity were identified and classified as low-, medium-, or high-intensity (details and definitions in Supplementary Table S1). Medications taken at discharge from the index admission and during the one-year follow-up were recorded in the KAMIR-NIH registry.

The primary endpoint was a two-year major adverse cardiac and cerebrovascular event (MACCE), defined as the composite of cardiovascular mortality, recurrent MI, and ischemic stroke since the 1-year follow-up visit (Figure 1a). All-cause mortality, any repeat revascularization, and hospitalization for heart failure were the secondary endpoints, as well as the single components of MACCE. All deaths were considered cardiovascular deaths unless an undisputed non-cardiovascular cause was identified. Recurrent MI was defined as elevated cardiac biomarkers with concomitant ischemic symptoms or electrocardiographic findings suggestive of ischemia [9]. Repeat revascularization was documented as clinically driven revascularization, including PCI or coronary artery bypass grafting (CABG) that occurred after discharge from the index admission.

### 2.4. Statistical Analysis

Categorical variables are reported as counts and percentages, while continuous variables are reported as medians with interquartile ranges. Group comparisons were performed using the Mann-Whitney test for continuous variables and the chi-square test for categorical variables. The difference in LDL-C level between the index admission and the one-year follow-up visit was calculated. To examine the relationship between the difference in LDL-C level as a continuous variable and the primary endpoint, a restricted cubic spline curve was plotted to explore the potential nonlinear relationship [10]. The cumulative incidences of MACCE are presented as Kaplan–Meier censoring estimates and analyzed with Cox proportional hazards models to calculate the hazard ratio (HR) and 95% confidence interval (95% CI). The multivariable Cox regression model included age, sex, body mass index, smoking, hypertension, diabetes, statin usage before the index event, history of PCI/CABG, chronic kidney disease, multivessel disease at the index event, left-main disease at the index event, presentation as ST-segment elevation MI (STEMI), LVEF < 40%, LDL-C level at the index MI, and complete revascularization after the index event.

Furthermore, the sub-distribution hazard ratio for the primary endpoint was estimated using the Fine-Gray competing risk model [11], and non-cardiovascular death was modelled as a single competing outcome. Subgroup analyses were also performed according to the presence of hypertension, diabetes, CKD, LVEF < 40%, presentation as STEMI, and high-intensity statin therapy at discharge from the index admission. Secondary analyses were performed to account for changes in LDL-C levels. Statistical analyses were performed using R statistical software (version 4.1.2; R Foundation for Statistical Computing, Vienna, Austria) with two-sided values of *p* < 0.05 considered statistically significant, except in the subgroup analysis, in which values of *p* < 0.1 were considered statistically significant.

## 3. Results

This prospective cohort included a total of 5049 patients in the final analysis, of whom 1114 (22.1%) reached the target LDL-C level at the one-year follow-up. The median LDL-C level at the index MI admission was 118 (92.6–114) mg/dL for all study subjects (Table 1), 121 (94.3–146) mg/dL for statin-naïve patients, and 98 (73.4–122) mg/dL for those with statin exposure before the index MI event. Direct LDL-C measurements were available in most cases, and 2.3% of all measurements (combined baseline and follow-up) used the calculated LDL-C level assessed using the Friedewald equation. At the one-year follow-up visit, 2303 (45.6%) patients achieved an LDL-C level ≤ 70 mg/dL (1.8 mmol/L) and 1438 (28.5%) patients had an LDL-C reduction ≥ 50% from baseline (Table 2). Overall, 22.1% (1114/5049) of all patients achieved both an LDL-C level ≤ 70 mg/dL and an LDL-C reduction ≥ 50% from baseline, and were classified as target LDL-C achievers.

### 3.1. Clinical Characteristics

The demographic and clinical characteristics at the index MI admission are presented in Table 1. The median age of the overall study population was 60 years, 21% of them were women, while 52.1% were diagnosed with STEMI. Comorbid conditions including dyslipidemia, previous MI, previous revascularization, previous stroke, and CKD were less frequent in target LDL-C achievers compared to non-achievers. The clinical presentation of index MI events showed comparable angiographic and procedural results, except for cardiogenic shock, which was higher in non-achievers.

### 3.2. Statin Exposure and LDL-C Reduction

Overall, 4547 (90.1%) patients were statin-naïve at the presentation of index MI events, and target LDL-C achievers included a higher proportion of statin-naïve patients (Supplementary Table S2). Almost all patients (98.3%) among the target LDL-C achievers were prescribed statins as discharge medication, and high-intensity statin therapy was more frequently prescribed to target achievers (Table 1). The median change in LDL-C was a 77 mg/dL reduction in target achievers, with a median LDL-C level of 53.4 mg/dL at the one-year follow-up (Table 2). A significant proportion (14.7%) of all patients showed no reduction or increase in LDL-C level at follow-up. (Supplementary Figure S1). Non-achievers were less likely to maintain ongoing statin therapy (90% vs. 93%), and 5.1% of non-achievers did not receive statin therapy at the one-year follow-up visit (Supplementary Table S2).

### 3.3. Clinical Outcomes

**Figure 2:**
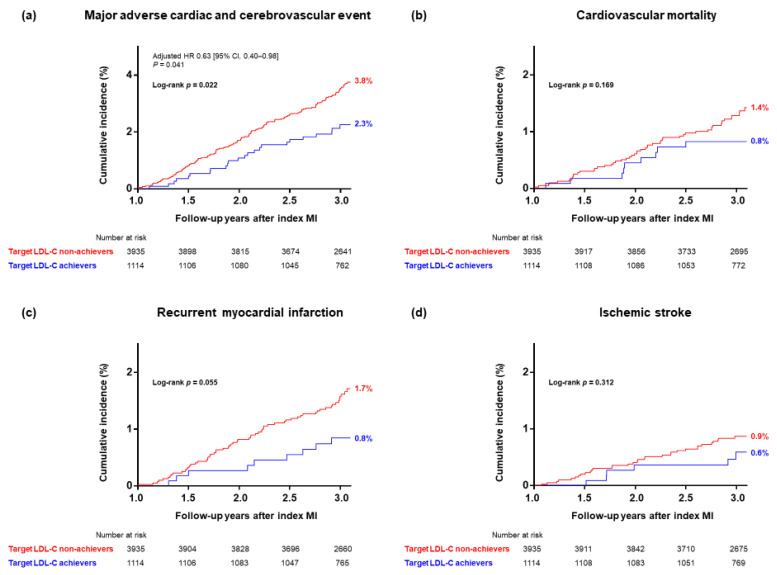
Cumulative incidence of (**a**) major adverse cardiac and cerebrovascular event, (**b**) cardiovascular mortality, (**c**) recurrent myocardial infarction, and (**d**) ischemic stroke according to the attainment of target LDL-C levels. CI, confidence interval; HR, hazard ratio; LDL-C, low-density lipoprotein cholesterol; MI, myocardial infarction.

**Figure 3:**
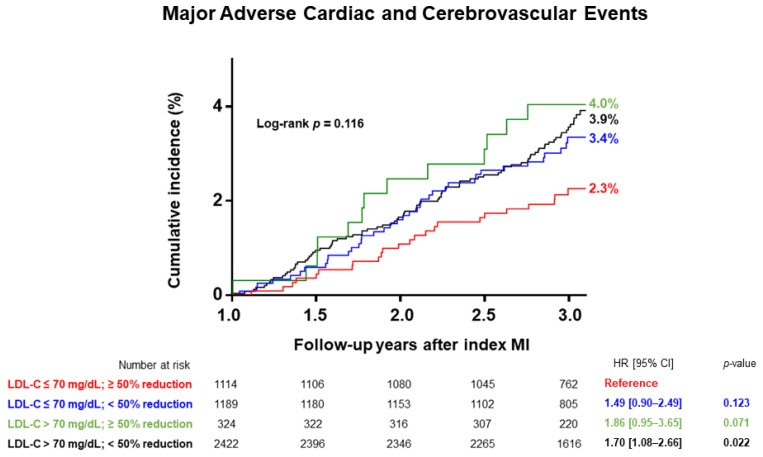
Cumulative incidence of the primary endpoint according to the achievement of either target LDL-C goal. CI, confidence interval; HR, hazard ratio; LDL-C, low-density lipoprotein cholesterol; MI, myocardial infarction.

After a median follow-up of 2.06 (1.97–2.14) years, 111 (2.2%) patients died of any cause, 71 (1.4%) had recurrent MI, and 39 (0.8%) had ischemic stroke. Table 3 summarizes the primary and secondary endpoints, while Figure 2 shows the cumulative incidence of MACCEs and its components since the one-year follow-up visit. Target LDL-C achievers demonstrated a significantly lower incidence (2.2% vs. 3.5%) and reduced adjusted hazard of MACCEs on the Cox regression model (0.63; 95% CI, 0.40–0.98). For those who received ongoing statin therapy at one-year follow-up but did not achieve the target LDL-C level ≤ 70 mg/dL, 20% (479/2444) had no reduction or even an increase in LDL-C at the one-year follow-up (Supplementary Table S3). In these patients, a ≥50% reduction in LDL-C was not significantly associated with a lower incidence of MACCE versus those with a 0–50% reduction in LDL-C (3.3% vs. 2.8%, *p* = 0.670). Figure 3 shows the cumulative incidence of MACCE according to the achievement of either target LDL-C goal divided into four mutually exclusive groups. For those who achieved a ≥50% reduction in LDL-C at the one-year follow-up, patients with a target LDL-C level ≤ 70 mg/dL demonstrated a numerically lower incidence of the primary endpoint (2.3% vs. 4.0%, log-rank test, *p* = 0.068). Thus, achieving a follow-up LDL-C level ≤ 70 mg/dL might be more important than obtaining a ≥50% reduction in LDL-C levels.

### 3.4. Secondary Analyses

To take changes in LDL-C decline into account, the Kaplan-Meier censoring estimates of MACCE were plotted according to the percent change in LDL-C (log-rank test, *p* < 0.001; Supplementary Figure S2). Of note, patients with ≥60% reduction in LDL-C showed a higher incidence of MACCE than those with a 40–60% reduction in LDL-C (4.0% vs. 2.3%, *p* = 0.024). The degree of LDL-C change showed a curvilinear relationship, whereas the LDL-C level at the one-year follow-up was linearly correlated with the hazard ratio of MACCE occurrence (Supplementary Figure S3).

### 3.5. Predictors of the Primary Endpoint

When non-cardiovascular death was modelled as a single competing outcome, the sub-distribution hazard ratio for MACCE by the Fine-Gray model was 0.63 (95% CI, 0.41–0.98; *p* = 0.041) for target LDL-C achievers (Supplementary Table S4). The independent predictors of MACCE assessed by the Cox regression model were age, diabetes, statin usage before the index MI event, history of PCI/CABG, CKD, LVEF < 40%, and target LDL-C achievement (Supplementary Table S5). CKD was the strongest predictor of MACCE (HR 2.49; 95% CI, 1.38–4.47). Statin usage before the index MI event and achievement of the target LDL-C goal at the one-year follow-up were associated with a decreased adjusted hazard for MACCE. In a subgroup analysis according to statin usage before the index event, target LDL-C achievement was associated with a lower risk of MACCEs in the statin-naïve subgroup (*P* for interaction = 0.661; Supplementary Figure S4).

## 4. Discussion

The current study evaluated the clinical impact of target LDL-C achievement on MACCE recurrence in patients with acute MI, using a prospective, multicenter, nationwide registry. The main findings of the present analysis included: (1) patients who achieved the LDL-C target goal at the one-year follow-up (both ≥50% reduction and LDL-C level ≤ 70 mg/dL), demonstrated a lower incidence and reduced adjusted hazard of MACCE, including cardiovascular mortality, recurrent MI, and ischemic stroke; (2) the relationship between LDL-C level at the one-year follow-up and hazard ratio of MACCE occurrence was linear, whereas the degree of LDL-C change was not; (3) although 95.5% of all study subjects were discharged with statin therapy and continued on the medication at the one-year follow-up visit (90.7% were ongoing; 4.6% were newly started), 2141 (42.4%) patients did not achieve both target goals (≥50% reduction and LDL-C level ≤ 70 mg/dL).

### 4.1. Achieving Target LDL-C Goal for Secondary Prevention following Acute MI

Recent updates on European and American guidelines recommend achieving an LDL-C percentage reduction to a minimum of 50% in patients at very high risk for recurrent atherosclerotic cardiovascular events [1,12]. Although the ESC/EAS guideline advocates targeting an LDL-C level < 55 mg/dL and a ≥50% LDL-C reduction in patients in very high-risk group, the AHA/ACC guideline use an LDL-C threshold of 70 mg/dL to consider the addition of non-statins to statin therapy in this very high-risk group. The Korean guideline recommends a target LDL-C level < 70 mg/dL or ≥50% reduction from the baseline level in patients with coronary artery diseases [13]. These therapeutic target goals of secondary prevention in the Korean population were previously examined in real-world settings. In the KAMIR study from February 2008 to November 2011, achieving a ≥50% reduction in LDL-C was associated with better clinical outcomes in 1305 patients after acute MI, whereas achieving a level < 70 mg/dL was not [14]. A follow-up study of 3315 patients from January 2008 to September 2012 showed that achieving a target LDL-C level < 70 mg/dL did not result in better clinical outcomes, as assessed by propensity-matched analysis [15]. Real-world data also revealed that achieving the target LDL-C level is suboptimal in Koreans, as only 39% of those at very high risk of a clinical event achieved target LDL-C levels [6]. In the present analysis, 2627 (52.0%) patients met the therapeutic target goals of the current Korean guidelines, which gave poor discrimination of hard endpoints versus those with both a <50% reduction and a follow-up LDL-C level > 70 mg/dL (3.0% vs. 3.9%, log-rank test, *p* = 0.121; Figure 3). Thus, achieving both a ≥50% reduction and an LDL-C level ≤ 70 mg/dL for secondary prevention is crucial for improving clinical outcomes in post-MI patients. Moreover, despite the high prevalence (98.4%; 4745/4824) of medium- or high-intensity statin therapy at discharge, 42.4% of all study subjects did not achieve both target goals. This emphasizes the variable response to statin treatment and residual risks, which corroborate the importance of add-on ezetimibe and/or PCSK9 inhibitors. This suboptimal attainment of the LDL-C reduction in the Korean population should be improved to prevent recurrent ischemic events in patients with acute MI.

### 4.2. Degree of LDL-C Reduction and Clinical Events

A previous large-scale study demonstrated a linear relationship between LDL-C reduction and clinical event rate decline [16]. Interestingly, we observed very similar population distribution of LDL-C absolute reduction and percent reductions from the index MI event, with the similar median change in LDL-C of 1.14 (0.44–1.84) mmol/L (Supplementary Figure S1). However, the degree of LDL-C change showed a curvilinear relationship with the hazard ratio of MACCE occurrence, whereas the absolute level of LDL-C at the one-year follow-up was linearly correlated. (Supplementary Figure S3). This can be partly explained by the small number of patients (65/5049) with both a ≥60% reduction and follow-up LDL-C level > 70 mg/dL whose baseline level of LDL-C level was 5.61 (5.16–6.05) mmol/L and the median change in LDL-C was 3.52 (3.24–3.93) mmol/L. These 65 subjects had a 6.2% two-year cumulative incidence of MACCE, comparable to the event rate of those with no reduction or increase in follow-up LDL-C levels (Supplementary Figure S2). Subsequently, patients (11.3%; 571/5049) with a ≥60% reduction may have skewed the dataset away from the linear relationship with the hazard of hard endpoints. Current cholesterol guidelines assume a log linear association between absolute LDL-C reduction and relative risk reduction in cardiovascular outcomes. Previous meta-analyses confirmed a dose-dependent reduction in clinical events with statin therapy based on between-group differences in achieved LDL-C levels [17,18,19,20]. Since these meta-analyses mostly included drug trials with an absolute LDL-C reduction ≤ 3 mmol/L, the results of the present analysis do not contradict previous data. From our real-world registry data, achieving a follow-up LDL-C level ≤ 70 mg/dL might be more important than obtaining a large percentage reduction in LDL-C levels (Figure 3). Therefore, in patients with higher baseline LDL-C levels, targeting the absolute LDL-C level rather than focusing on percentage reduction might help physicians to improve clinical outcomes.

### 4.3. Limitations

This prospective observational study design has some limitations. First, a substantial number of patients in the KAMIR-NIH registry were excluded, and our study only included patients whose LDL-C levels were available with relatively short periods of clinical follow-up, limiting the representativeness of the general population with acute MI. Second, information on LDL-C-lowering agents other than statins was unavailable. As the study subjects were enrolled between November 2011 and December 2015, PCSK9 inhibitors were not commercially available in South Korea at that time, and the number of ezetimibe prescriptions as an add-on to statin were very limited (5.2%; 251/4824; data not shown). Finally, because we only analyzed LDL-C changes, the relationship between high-density lipoprotein cholesterol and triglycerides levels with hard endpoints remains to be determined.

## 5. Conclusions

In patients with acute MI, achieving a ≥50% reduction and follow-up LDL-C level ≤ 70 mg/dL at the one-year follow-up were associated with lower incidence and a reduced hazard of recurrent clinical events. Our results highlight the need to improve current practices in managing LDL-C levels for secondary prevention and the importance of applying new guidelines, including add-on nonstatins, in this very high-risk populations.

## Figures and Tables

**Table 1:** Clinical characteristics at index myocardial infarction admission.

Characteristics	Overall (N = 5049)	Non-Achiever (*n* = 3935)	Achiever (*n* = 1114)	*p*-Value
**Demographics**				
Age, year	60.0 [52.0; 70.0]	61.0 [52.0; 70.0]	60.0 [52.0; 70.0]	0.204
Female sex	1060 (21.0%)	872 (22.2%)	188 (16.9%)	<0.001
BMI > 23 kg/m^2^	3455 (68.4%)	2657 (67.5%)	798 (71.6%)	0.010
**Medical history**				
Current smoker	2226 (44.1%)	1710 (43.5%)	516 (46.3%)	0.096
Hypertension	2349 (46.5%)	1854 (47.1%)	495 (44.4%)	0.121
Diabetes	1314 (26.0%)	1031 (26.2%)	283 (25.4%)	0.620
Dyslipidemia	708 (14.0%)	615 (15.6%)	93 (8.3%)	<0.001
History of MI	257 (5.1%)	240 (6.1%)	17 (1.5%)	<0.001
History of PCI/CABG	203 (4.0%)	184 (4.7%)	19 (1.7%)	<0.001
History of HF	43 (0.9%)	39 (1.0%)	4 (0.4%)	0.066
History of CVA	235 (4.7%)	198 (5.0%)	37 (3.3%)	0.021
Chronic kidney disease	109 (2.2%)	92 (2.3%)	17 (1.5%)	0.126
**Laboratory variables**				
LDL-C (mg/dL)	118.0 [92.6; 144.0]	112.2 [87.0; 140.9]	133.0 [116.0; 150.0]	<0.001
HDL-C (mg/dL)	42.0 [35.0; 49.0]	41.0 [35.0; 49.0]	43.0 [36.2; 50.0]	0.002
Triglyceride (mg/dL)	112.0 [76.0; 172.0]	111.0 [74.0; 172.0]	116.0 [83.0; 172.0]	0.025
Total cholesterol (mg/dL)	180.0 [154.0; 210.0]	175.0 [148.0; 207.0]	195.0 [175.5; 217.0]	<0.001
hs-CRP (mg/L)	0.8 [0.0; 3.1]	0.7 [0.0; 3.0]	1.0 [0.0; 3.6]	0.022
HbA1c (%)	5.9 [5.6; 6.8]	5.9 [5.5; 6.8]	5.9 [5.6; 6.9]	0.213
LVEF < 40%	560 (11.1%)	454 (11.5%)	106 (9.5%)	0.065
**Clinical presentation**				
STEMI	2632 (52.1%)	2039 (51.8%)	593 (53.2%)	0.423
Multivessel disease	2251 (44.6%)	1757 (44.7%)	494 (44.3%)	0.883
LM disease	200 (4.0%)	166 (4.2%)	34 (3.1%)	0.094
Cardiogenic shock	302 (6.0%)	255 (6.5%)	47 (4.2%)	0.006
Newly developed HF	141 (2.8%)	114 (2.9%)	27 (2.4%)	0.457
Infarct-related artery				
Left main	86 (1.7%)	74 (1.9%)	12 (1.1%)	
Left anterior descending	2263 (44.8%)	1720 (43.7%)	543 (48.7%)	
Left circumflex	862 (17.1%)	662 (16.8%)	200 (18.0%)	
Right	1605 (31.8%)	1277 (32.5%)	328 (29.4%)	
**PCI results**				
Culprit-only	1423 (28.2%)	1098 (27.9%)	325 (29.2%)	0.427
Complete revascularization	3381 (67.0%)	2625 (66.7%)	756 (67.9%)	0.492
**Discharge medication**				
Statin	4824 (95.5%)	3729 (94.8%)	1095 (98.3%)	<0.001
No therapy	225 (4.5%)	206 (5.2%)	19 (1.7%)	
Low-intensity	79 (1.6%)	64 (1.6%)	15 (1.3%)	
Medium-intensity	2988 (59.2%)	2480 (63.0%)	508 (45.6%)	
High-intensity	1757 (34.8%)	1185 (30.1%)	572 (51.3%)	
Aspirin	5042 (99.9%)	3930 (99.9%)	1112 (99.8%)	1.000
Clopidogrel	3694 (73.2%)	2935 (74.6%)	759 (68.1%)	<0.001
Prasugrel	647 (12.8%)	518 (13.2%)	129 (11.6%)	0.178
Ticagrelor	1362 (36.3%)	978 (34.1%)	384 (43.3%)	<0.001
Beta-blocker	4388 (86.9%)	3415 (86.8%)	973 (87.3%)	0.662
ACEi/ARB	4097 (81.1%)	3183 (80.9%)	914 (82.0%)	0.407
CCB	355 (7.0%)	291 (7.4%)	64 (5.7%)	0.066

Values are presented as median [interquartile range] or *n* (%). ACEi, angiotensin-converting enzyme inhibitors; ARB, angiotensin receptor blocker; BMI, body mass index; CABG, coronary artery bypass graft surgery; CCB, calcium channel blocker; CVA, cerebrovascular accident; HbA1c, hemoglobin A1c; HDL-C, high-density lipoprotein cholesterol; HF, heart failure; hs-CRP, high-sensitivity C-reactive protein; LDL-C, low-density lipoprotein cholesterol; LM, left main; LVEF, left ventricular ejection fraction; MI, myocardial infarction; PCI, percutaneous coronary intervention; STEMI, ST-segment elevation MI.

**Table 2:** Laboratory findings and medications at one-year follow-up.

Characteristics	Overall (N = 5049)	Non-Achiever (*n* = 3935)	Achiever (*n* = 1114)	*p*-Value
**Laboratory variables**				
LDL-C (mg/dL)	73.0 [59.0; 90.0]	80.0 [67.0; 95.0]	53.4 [45.2; 61.0]	<0.001
LDL-C reduction (mg/dL)	44.2 [17.0; 71.0]	33.0 [8.0; 55.0]	77.0 [65.0; 93.0]	<0.001
LDL-C ≤ 70 mg/dL	2303 (45.6%)	1189 (30.2%)	1114 (100.0%)	<0.001
≥50% LDL-C reduction	1438 (28.5%)	324 (8.2%)	1114 (100.0%)	<0.001
HDL-C (mg/dL)	43.0 [37.0; 51.0]	43.0 [37.0; 51.0]	42.0 [35.0; 50.0]	<0.001
Triglyceride (mg/dL)	116.0 [83.0; 166.0]	118.0 [85.0; 171.0]	104.5 [76.0; 148.0]	<0.001
Total cholesterol (mg/dL)	136.0 [118.0; 157.0]	144.0 [127.0; 163.0]	114.0 [103.0; 125.0]	<0.001
hs-CRP (mg/L)	0.8 [0.3; 2.1]	0.8 [0.3; 2.1]	0.8 [0.3; 2.0]	0.820
HbA1c (%)	6.2 [5.7; 7.0]	6.2 [5.7; 7.0]	6.2 [5.8; 7.1]	0.198
LVEF < 40%	191 (6.5%)	159 (7.1%)	32 (4.5%)	0.017
**On-going medications**				
Statin	4580 (90.7%)	3541 (90.0%)	1039 (93.3%)	<0.001
Aspirin	4380 (86.7%)	3427 (87.1%)	953 (85.5%)	0.314
Clopidogrel	2009 (39.8%)	1563 (39.7%)	446 (40.0%)	0.140
Prasugrel	162 (3.2%)	141 (3.6%)	21 (1.9%)	0.067
Ticagrelor	232 (4.6%)	157 (4.0%)	75 (6.7%)	<0.001
Beta-blocker	3774 (74.7%)	2942 (74.8%)	832 (74.7%)	0.584
ACEi/ARB	2660 (52.7%)	2066 (52.5%)	594 (53.3%)	0.654
CCB	382 (7.6%)	310 (7.9%)	72 (6.5%)	0.386

Values are presented as median [interquartile range] or *n* (%). ACEi, angiotensin-converting enzyme inhibitors; ARB, angiotensin receptor blocker; CCB, calcium channel blocker; HbA1c, hemoglobin A1c; HDL-C, high-density lipoprotein cholesterol; hs-CRP, high-sensitivity C-reactive protein; LDL-C, low-density lipoprotein cholesterol; LVEF, left ventricular ejection fraction.

**Table 3:** Incidence and risk of primary and secondary endpoints.

	Non-Achiever (*n* = 3935)	Achiever (*n* = 1114)	Log-Rank *p*-Value	Adjusted HR * [95% CI]	*p*-Value
**Primary Endpoint**					
MACCE	139 (3.5%)	24 (2.2%)	0.022	0.63 [0.40–0.98]	0.041
**Secondary Endpoints**					
All-cause mortality	93 (2.4%)	18 (1.6%)	0.140	0.77 [0.46–1.31]	0.339
Cardiovascular mortality	52 (1.3%)	9 (0.8%)	0.169	0.70 [0.33–1.45]	0.334
Recurrent MI	62 (1.6%)	9 (0.8%)	0.055	0.48 [0.24–0.98]	0.044
Ischaemic stroke	33 (0.8%)	6 (0.5%)	0.312	0.67 [0.28–1.64]	0.384
Repeat revascularization	145 (3.7%)	34 (3.1%)	0.311	0.81 [0.56–1.19]	0.290
Hospitalization for HF	56 (1.4%)	9 (0.8%)	0.109	0.82 [0.39–1.73]	0.606

MACCE was defined as a composite of cardiovascular mortality, recurrent myocardial infarction, and ischemic stroke. * Adjusted for age, sex, body mass index, smoking, hypertension, diabetes, statin usage before the index event, history of percutaneous coronary intervention/coronary artery bypass grafting, chronic kidney disease, multivessel disease at the index event, left main disease at the index event, presentation as ST-segment elevation myocardial infarction, left ventricular ejection fraction < 40%, low-density lipoprotein cholesterol level at the index event (mmol/L), and complete revascularization after the index event. CI, confidence interval; HF, heart failure; MACCE, major adverse cardiac and cerebrovascular event; MI, myocardial infarction.

## Data Availability

The data generated in this study are available from the corresponding author upon reasonable request.

## Supplementary Materials

The following supporting information can be downloaded at: https://www.mdpi.com/article/10.3390/jcm11092650/s1, Figure S1: Distribution of absolute changes in LDL-C from the index event. Median and interquartile values are 1.1 [0.4–1.8] mmol/L (dotted lines); Figure S2: Cumulative incidence of the primary endpoint by percentage changes in LDL-C from the index event; Figure S3: (A) Spline curve for the association of absolute changes in LDL-C with the risk of MACCE. Median and interquartile range are presented as dotted lines. (B) Spline curve for the association of follow-up LDL-C level with the risk of MACCE. Target LDL-C goal of 70 mg/dL is presented as a dotted line; Figure S4: Subgroup analysis of target LDL-C achievement for the primary endpoint; Table S1: Statin intensity classification; Table S2: Statin exposure and LDL-C reduction; Table S3: Patients with ongoing statin therapy and an LDL-C level > 70 mg/dL at the 1-year follow-up visit; Table S4: Crude and adjusted hazard ratios of the primary endpoint; Table S5: Cox’s proportional hazard model analysis of the primary endpoint.

## References

[1] Mach F., Baigent C., Catapano A.L., Koskinas K.C., Casula M., Badimon L., Chapman M.J., De Backer G.G., Delgado V., Ference B.A. (2020). 2019 ESC/EAS Guidelines for the management of dyslipidaemias: Lipid modification to reduce cardiovascular risk. Eur. Heart J..

[2] Szummer K., Wallentin L., Lindhagen L., Alfredsson J., Erlinge D., Held C., James S., Kellerth T., Lindahl B., Ravn-Fischer A. (2017). Improved outcomes in patients with ST-elevation myocardial infarction during the last 20 years are related to implementation of evidence-based treatments: Experiences from the SWEDEHEART registry 1995–2014. Eur. Heart J..

[3] Schwartz G.G., Olsson A.G., Ezekowitz M.D., Ganz P., Oliver M.F., Waters D., Zeiher A., Chaitman B.R., Leslie S., Stern T. (2001). Effects of atorvastatin on early recurrent ischemic events in acute coronary syndromes: The MIRACL study: A randomized controlled trial. JAMA.

[4] de Lemos J.A., Blazing M.A., Wiviott S.D., Lewis E.F., Fox K.A., White H.D., Rouleau J.L., Pedersen T.R., Gardner L.H., Mukherjee R. (2004). Early intensive vs a delayed conservative simvastatin strategy in patients with acute coronary syndromes: Phase Z of the A to Z trial. JAMA.

[5] Gitt A.K., Lautsch D., Ferrières J., De Ferrari G.M., Vyas A., Baxter C.A., Bash L.D., Ashton V., Horack M., Almahmeed W. (2017). Cholesterol target value attainment and lipid-lowering therapy in patients with stable or acute coronary heart disease: Results from the Dyslipidemia International Study II. Atherosclerosis.

[6] Yang Y.S., Lee S.Y., Kim J.S., Choi K.M., Lee K.W., Lee S.C., Cho J.R., Oh S.J., Kim J.H., Choi S.H. (2020). Achievement of LDL-C Targets Defined by ESC/EAS (2011) Guidelines in Risk-Stratified Korean Patients with Dyslipidemia Receiving Lipid-Modifying Treatments. Endocrinol. Metab..

[7] Kim J.H., Chae S.C., Oh D.J., Kim H.S., Kim Y.J., Ahn Y., Cho M.C., Kim C.J., Yoon J.H., Park H.Y. (2016). Multicenter Cohort Study of Acute Myocardial Infarction in Korea–Interim Analysis of the Korea Acute Myocardial Infarction Registry-National Institutes of Health Registry. Circ. J..

[8] Krishnaveni P., Gowda V.M. (2015). Assessing the Validity of Friedewald’s Formula and Anandraja’s Formula For Serum LDL-Cholesterol Calculation. J. Clin. Diagn. Res..

[9] Moussa I.D., Klein L.W., Shah B., Mehran R., Mack M.J., Brilakis E.S., Reilly J.P., Zoghbi G., Holper E., Stone G.W. (2013). Consideration of a new definition of clinically relevant myocardial infarction after coronary revascularization: An expert consensus document from the Society for Cardiovascular Angiography and Interventions (SCAI). J. Am. Coll. Cardiol..

[10] Heinzl H., Kaider A. (1997). Gaining more flexibility in Cox proportional hazards regression models with cubic spline functions. Comput. Methods Programs Biomed..

[11] Austin P.C., Lee D.S., Fine J.P. (2016). Introduction to the Analysis of Survival Data in the Presence of Competing Risks. Circulation.

[12] Grundy S.M., Stone N.J., Bailey A.L., Beam C., Birtcher K.K., Blumenthal R.S., Braun L.T., de Ferranti S., Faiella-Tommasino J., Forman D.E. (2019). 2018 AHA/ACC/AACVPR/AAPA/ABC/ACPM/ADA/AGS/APhA/ASPC/NLA/PCNA Guideline on the Management of Blood Cholesterol: A Report of the American College of Cardiology/American Heart Association Task Force on Clinical Practice Guidelines. Circulation.

[13] Rhee E.J., Kim H.C., Kim J.H., Lee E.Y., Kim B.J., Kim E.M., Song Y., Lim J.H., Kim H.J., Choi S. (2019). 2018 Guidelines for the management of dyslipidemia. Korean J. Intern. Med..

[14] Cho K.H., Jeong M.H., Park K.W., Kim H.S., Lee S.R., Chae J.K., Hong Y.J., Kim J.H., Ahn Y., Cho J.G. (2015). Comparison of the effects of two low-density lipoprotein cholesterol goals for secondary prevention after acute myocardial infarction in real-world practice: ≥50% reduction from baseline versus <70 mg/dL. Int. J. Cardiol..

[15] Ahn T., Suh S.Y., Lee K., Kang W.C., Han S.H., Ahn Y., Jeong M.H. (2017). Clinical Outcomes according to the Achievement of Target Low Density Lipoprotein-Cholesterol in Patients with Acute Myocardial Infarction. Korean Circ. J..

[16] Schubert J., Lindahl B., Melhus H., Renlund H., Leosdottir M., Yari A., Ueda P., James S., Reading S.R., Dluzniewski P.J. (2021). Low-density lipoprotein cholesterol reduction and statin intensity in myocardial infarction patients and major adverse outcomes: A Swedish nationwide cohort study. Eur. Heart J..

[17] Cholesterol Treatment Trialists C., Baigent C., Blackwell L., Emberson J., Holland L.E., Reith C., Bhala N., Peto R., Barnes E.H., Keech A. (2010). Efficacy and safety of more intensive lowering of LDL cholesterol: A meta-analysis of data from 170,000 participants in 26 randomised trials. Lancet.

[18] Mihaylova B., Emberson J., Blackwell L., Keech A., Simes J., Barnes E.H., Voysey M., Gray A., Collins R., Cholesterol Treatment Trialists’ (CTT) Collaborators (2012). The effects of lowering LDL cholesterol with statin therapy in people at low risk of vascular disease: Meta-analysis of individual data from 27 randomised trials. Lancet.

[19] Fulcher J., O’Connell R., Voysey M., Emberson J., Blackwell L., Mihaylova B., Simes J., Collins R., Kirby A., Cholesterol Treatment Trialists’ (CTT) Collaborators (2015). Efficacy and safety of LDL-lowering therapy among men and women: Meta-analysis of individual data from 174,000 participants in 27 randomised trials. Lancet.

[20] Silverman M.G., Ference B.A., Im K., Wiviott S.D., Giugliano R.P., Grundy S.M., Braunwald E., Sabatine M.S. (2016). Association Between Lowering LDL-C and Cardiovascular Risk Reduction Among Different Therapeutic Interventions: A Systematic Review and Meta-analysis. JAMA.

