# Supplementary material for: Target Low-Density Lipoprotein-Cholesterol and Secondary Prevention for Patients with Acute Myocardial Infarction: A Korean Nationwide Cohort Study
Source: J Clin Med. 2022 May 8;11(9):2650. doi: 10.3390/jcm11092650 (PMC9104536; doi:10.3390/jcm11092650)

Supplementary Materials

I. Supplementary Tables

Table S1. Statin intensity classification

Table S2. Statin exposure and LDL-C reduction

Table S3. Patients with ongoing statin therapy and an LDL-C level > 70 mg/dL at the 1-year follow-up visit

Table S4. Crude and adjusted hazard ratios of the primary endpoint

Table S5. Cox's proportional hazard model analysis of the primary endpoint

II. Supplementary Figures

Figure S1. Distribution of absolute changes in LDL-C from the index event

Figure S2. Cumulative incidence of the primary endpoint by percentage changes in LDL-C from the index event.

Figure S3. (A) Spline curve for the association of absolute changes in LDL-C with the risk of MACCE. (B) Spline curve for the association of follow-up LDL-C level with the risk of MACCE.

Figure S4. Subgroup analysis of target LDL-C achievement for the primary endpoint.

Table S1. Statin intensity classification.

Statin	Low-	Medium-	High-
Atorvastatin	-	10 ~ <40 mg	≥40 mg
Rosuvastatin	-	5 ~ <20 mg	≥20 mg
Simvastatin	<20 mg	20 ~ <80mg	80 mg
Pitavastatin	1 mg	2 ~ 4 mg	-
Pravastatin	<40 mg	40 ~ 80 mg	-
Fluvastatin	≤40 mg	80 mg	

Table S2. Statin exposure and LDL-C reduction.

Characteristics	Overall (N = 5,049)	Non-achiever (n = 3,935)	Achiever (n = 1,114)	*P* value
Index MI admission				
LDL-C (mmol/L)	3.1 [2.4; 3.7]	2.9 [2.2; 3.6]	3.4 [3.0; 3.9]	<0.001
Statin-naïve at presentation	4547 (90.1%)	3481 (88.5%)	1066 (95.7%)	<0.001
Statin at discharge				
Atorvastatin	2340 (46.3%)	1863 (47.3%)	477 (42.8%)	
Rosuvastatin	1944 (38.5%)	1398 (35.5%)	546 (49.0%)	
Simvastatin	281 (5.6%)	234 (5.9%)	47 (4.2%)	
Pitavastatin	210 (4.2%)	188 (4.8%)	22 (2.0%)	
Pravastatin	45 (0.9%)	42 (1.1%)	3 (0.3%)	
Fluvastatin	4 (0.1%)	4 (0.1%)	0 (0.0%)	
One-year follow-up visit				
LDL-C (mmol/L)	1.9 [1.5; 2.3]	2.1 [1.7; 2.5]	1.4 [1.2; 1.6]	<0.001
LDL-C reduction (mmol/L)	1.1 [0.4; 1.8]	0.9 [0.2; 1.4]	2.0 [1.7; 2.4]	<0.001
No reduction or increase	740 (14.7%)	740 (18.8%)	0 (0.0%)	
0 - 50% reduction	2871 (56.9%)	2871 (73.0%)	0 (0.0%)	
≥ 50% reduction	1438 (28.5%)	324 (8.2%)	1114 (100.0%)	
Statin				
On-going	4580 (90.7%)	3541 (90.0%)	1039 (93.3%)	<0.001
Newly started	234 (4.6%)	186 (4.7%)	48 (4.3%)	
Stopped	102 (2.0%)	82 (2.1%)	20 (1.8%)	

No use	121 (2.4%)	117 (3.0%)	4 (0.4%)	
Statin during follow-up				
On-going at 2-year visit	4545 (90.0%)	3513 (89.3%)	1032 (92.6%)	0.008
On-going at 3-year visit	4363 (86.4%)	3374 (85.7%)	989 (88.8%)	0.001

Values are presented as median [interquartile range] or n (%). LDL-C, low-density lipoprotein cholesterol.

Table S3. Patients with ongoing statin therapy and an LDL-C level > 70 mg/dL at the 1-year follow-up visit.

Characteristics	No reduction or increase (n = 479)	0-50% reduction (n = 1,662)	≥ 50% reduction (n = 303)	*P* value
**Demographics**				
Age, year	62.0 [53.0;72.0]	59.0 [51.0;69.0]	56.0 [47.5;65.0]	<0.001
Female sex	126 (26.3%)	371 (22.3%)	73 (24.1%)	0.182
BMI > 23 kg/m^2	328 (68.5%)	1149 (69.1%)	212 (70.0%)	0.907
Current smoker	178 (37.2%)	787 (47.4%)	143 (47.2%)	<0.001
Hypertension	286 (59.7%)	730 (43.9%)	96 (31.7%)	<0.001
Diabetes	181 (37.8%)	312 (18.8%)	45 (14.9%)	<0.001
Statin-naïve at presentation	360 (75.2%)	1502 (90.4%)	290 (95.7%)	<0.001
History of PCI/CABG	66 (13.8%)	29 (1.7%)	6 (2.0%)	<0.001
Chronic kidney disease	16 (3.3%)	25 (1.5%)	5 (1.7%)	0.032
**Clinical presentation**				
STEMI	215 (44.9%)	897 (54.0%)	164 (54.1%)	0.002
Multivessel disease	216 (45.1%)	764 (46.0%)	126 (41.6%)	0.369
LM disease	31 (6.5%)	63 (3.8%)	16 (5.3%)	0.035
LVEF <40%	64 (13.4%)	177 (10.6%)	24 (7.9%)	0.053
Complete revascularization	291 (60.8%)	1131 (68.1%)	217 (71.6%)	0.002
**Statin therapy at discharge**				**<0.001**
Low-intensity	12 (2.5%)	24 (1.4%)	2 (0.7%)	

Medium-intensity	364 (76.0%)	1139 (68.5%)	131 (43.2%)	
High-intensity	86 (18.0%)	485 (29.2%)	168 (55.4%)	
**LDL-C levels**				
Baseline (mmol/L)	2.1 [1.8; 2.5]	3.4 [2.9; 3.9]	4.7 [4.2; 5.2]	<0.001
1-year follow-up (mmol/L)	2.6 [2.2; 3.1]	2.2 [2.0; 2.6]	2.0 [1.9; 2.2]	<0.001
Reduction (mmol/L)	-0.4 [-0.8;-0.2]	1.0 [0.6; 1.6]	2.6 [2.3; 3.0]	<0.001
**Clinical outcomes***				
MACCE	25 (5.2%)	47 (2.8%)	10 (3.3%)	0.033
Cardiovascular mortality	7 (1.5%)	15 (0.9%)	2 (0.7%)	0.439
Recurrent MI	10 (2.1%)	25 (1.5%)	5 (1.7%)	0.649
Ischemic stroke	9 (1.9%)	8 (0.5%)	4 (1.3%)	0.009

Values are presented as median [interquartile range] or n (%). BMI, body mass index; CABG, coronary artery bypass graft surgery; HF, heart failure; LDL-C, low-density lipoprotein cholesterol; LM, left-main; LVEF, left ventricular ejection fraction; MACCE, major adverse cardiac and cerebrovascular events; MI, myocardial infarction; PCI, percutaneous coronary intervention; STEMI, ST-segment elevation MI. *The log-rank test *p*-values are presented for clinical outcomes comparing the three groups according to the percentage reduction in LDL-C.

Table S4. Crude and adjusted hazard ratios of the primary endpoint.

Target LDL-C achiever (vs. non-achiever)	Crude Hazard Ratio (95% CI)	*P*-value	*Adjusted Hazard Ratio (95% CI)	*P*-value
Cox proportional hazard model	0.61 [0.39 - 0.94]	0.024	0.63 [0.40 - 0.98]	0.041
Competing risks†	0.61 [0.40 - 0.94]	0.026	0.63 [0.41 - 0.98]	0.041

*Adjusted for age, sex, body mass index, smoking, hypertension, diabetes, statin usage before index event, history of PCI/CABG, chronic kidney disease, multivessel disease at index event, left-main disease at index event, presentation as STEMI, LV ejection fraction < 40%, LDL-C at index event (mmol/L), and complete revascularization after index event. †Non-cardiovascular death was modelled as a single competing outcome by using the Fine-Gray method. CI, confidence interval.

Table S5. Cox's proportional hazard model analysis of the primary endpoint.

Variables	Univariable HR (95% CI)	*P*-value	Multivariable HR (95% CI)	*P*-value
Age (per years)	1.04 [1.02 - 1.05]	<0.001	1.03 [1.01 - 1.04]	0.002
Female	1.40 [0.99 - 1.98]	0.056	0.98 [0.67 - 1.44]	0.920
Body mass index > 23 kg/m^2	0.65 [0.48 - 0.89]	0.008	0.77 [0.56 - 1.07]	0.120
Hypertension	1.62 [1.18 - 2.21]	0.003	1.15 [0.82 - 1.62]	0.413
Diabetes	2.36 [1.73 - 3.21]	<0.001	1.90 [1.36 - 2.66]	< 0.001
Smoking	0.65 [0.47 - 0.90]	0.010	1.02 [0.70 - 1.49]	0.900
Chronic kidney disease	4.77 [2.76 - 8.25]	<0.001	2.49 [1.38 - 4.47]	0.002
Previous PCI/CABG	3.32 [2.06 - 5.36]	<0.001	2.26 [1.34 - 3.81]	0.002
Statin usage before index MI	0.65 [0.35 - 1.20]	0.168	0.46 [0.24 - 0.86]	0.015
LVEF < 40%	2.50 [1.74 - 3.59]	<0.001	1.90 [1.30 - 2.76]	< 0.001
Presentation as STEMI	0.83 [0.61 - 1.13]	0.247	1.03 [0.75 - 1.43]	0.834
Left-main disease	1.76 [0.96 - 3.25]	0.070	1.13 [0.56 - 2.29]	0.730
Multivessel disease	1.52 [1.12 - 2.07]	0.008	1.18 [0.79 - 1.77]	0.410
Complete revascularization	0.58 [0.43 - 0.79]	<0.001	0.80 [0.54 - 1.17]	0.251

High-intensity statin at discharge	0.83 [0.60 - 1.16]	0.284	0.98 [0.70 - 1.38]	0.904
LDL-C at index admission (mmol/L)	0.83 [0.71 - 0.98]	0.025	1.05 [0.89 - 1.24]	0.535
Target LDL-C achievement	0.61 [0.39 - 0.94]	0.024	0.63 [0.40 - 0.99]	0.044

MI, myocardial infarction; CABG, coronary artery bypass graft surgery; PCI, percutaneous coronary intervention; LDL-C, low-density lipoprotein cholesterol; LVEF, left ventricular ejection fraction; STEMI, ST-segment elevation MI.

Figure S1. Distribution of absolute changes in LDL-C from the index event. Median and interquartile values are 1.1 [0.4 - 1.8] mmol/L (dotted lines). LDL-C, low-density lipoprotein cholesterol; MI, myocardial infarction

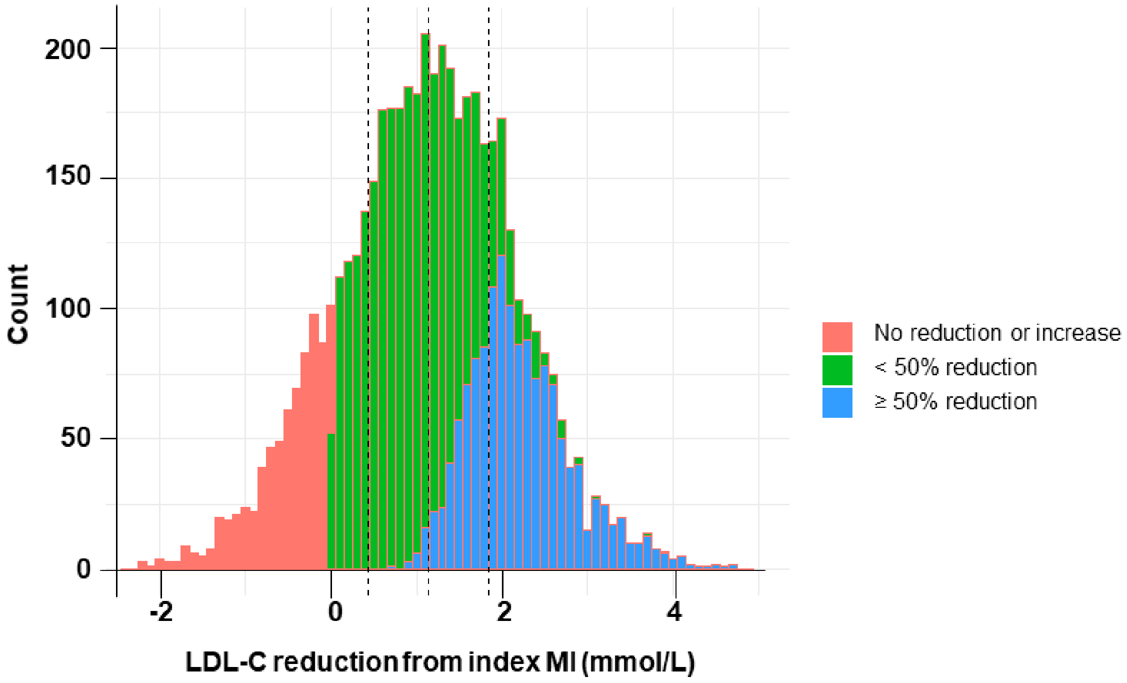

Figure S2. Cumulative incidence of the primary endpoint by percentage changes in LDL-C from the index event. LDL-C, low-density lipoprotein cholesterol; MI, myocardial infarction

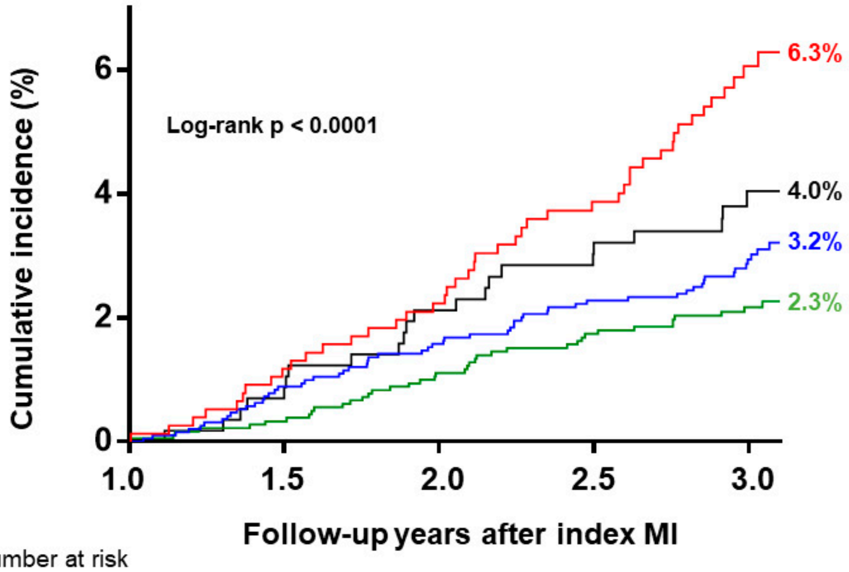

Number at risk					
No reduction or increase	766	755	733	692	489
0 - 40% reduction	1,907	1,888	1,850	1,798	1,292
40 – 60% reduction	1,805	1,798	1,764	1,700	1,231
≥ 60% reduction	571	563	548	529	391

Figure S3. (A) Spline curve for the association of absolute changes in LDL-C with the risk of MACCE. Median and interquartile range are presented as dotted lines. (B) Spline curve for the association of follow-up LDL-C level with the risk of MACCE. Target LDL-C goal of 70 mg/dL is presented as a dotted line. LDL-C, low-density lipoprotein cholesterol; MACCE, major adverse cardiac and cerebrovascular event; MI, myocardial infarction

(A)

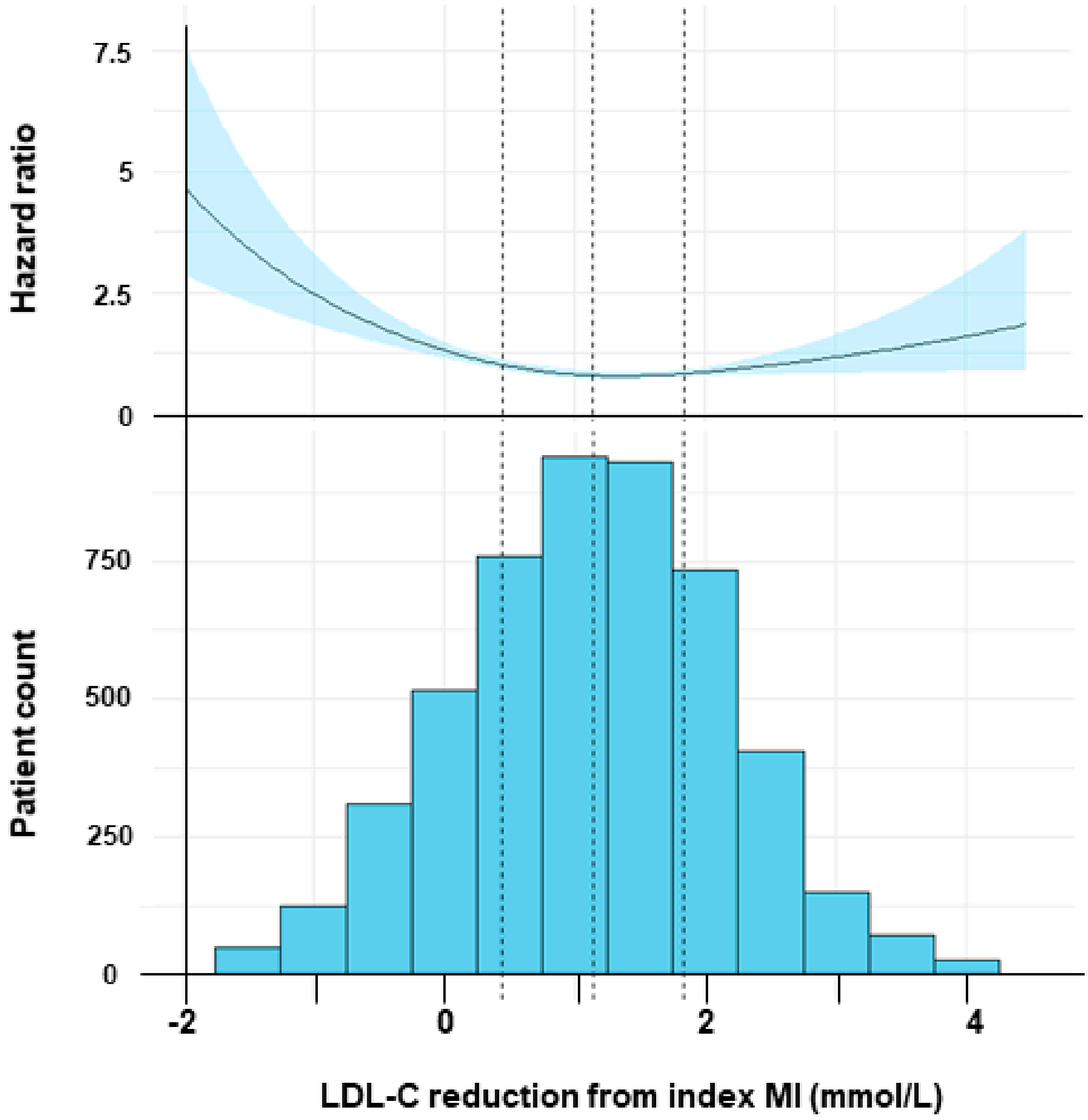

(B)

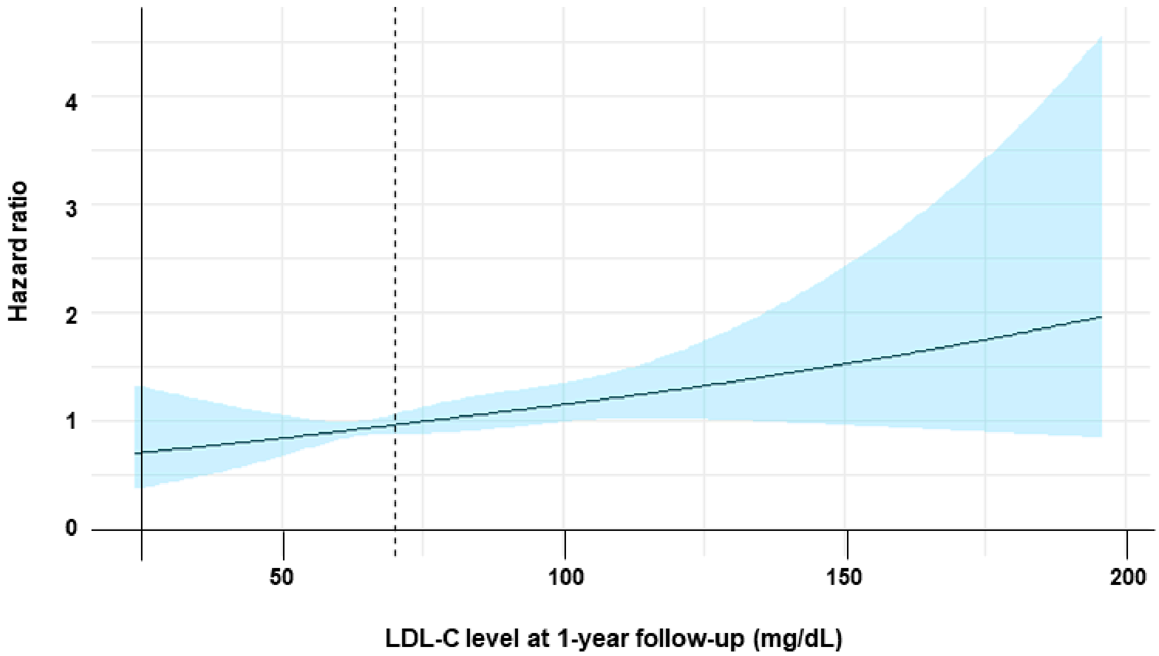

Hazard ratio
0
1
2
3
4
50
100
150
200
LDL-C level at 1-year follow-up (mg/dL)

Figure S4. Subgroup analysis of target LDL-C achievement for the primary endpoint. CI, confidence interval; CKD, chronic kidney disease; LDL-C, low-density lipoprotein cholesterol; LVEF, left ventricular ejection fraction.

Subgroup		No. of Patients	Non-achievers (N=3,935)	Achievers (N=1,114)	Hazard Ratio (95% CI)	P value for Interaction
			No of patients with event (%)			
Overall		5,049	139 (3.5%)	24 (2.2%)	0.61 [0.39 - 0.94]	
Diabetes						0.783
	Yes	1,314	63 (6.1%)	10 (3.5%)	0.57 [0.29 - 1.11]	
	No	3,735	76 (2.6%)	14 (1.7%)	0.64 [0.36 - 1.14]	
CKD						0.658
	Yes	109	12 (13.0%)	2 (11.8%)	0.88 [0.20 - 3.92]	
	No	4,940	127 (3.3%)	22 (2.0%)	0.60 [0.38 - 0.95]	
Statin-naïve						0.661
	Yes	4,547	129 (3.7%)	23 (2.2%)	0.58 [0.37 - 0.90]	
	No	502	10 (2.2%)	1 (2.1%)	0.94 [0.12 - 7.37]	
LVEF < 40%						0.971
	Yes	560	33 (7.3%)	5 (4.7%)	0.63 [0.25 - 1.62]	
	No	4,489	106 (3.0%)	19 (1.9%)	0.62 [0.38 - 1.01]	
STEMI						0.937
	Yes	2,632	66 (3.2%)	12 (2.0%)	0.62 [0.33 - 1.15]	
	No	2,417	73 (3.9%)	12 (2.3%)	0.60 [0.32 - 1.10]	
High-intensity statin						0.492
	Yes	1,757	40 (3.4%)	10 (1.7%)	0.52 [0.26 - 1.04]	
	No	3,292	99 (3.6%)	14 (2.6%)	0.71 [0.40 - 1.24]	

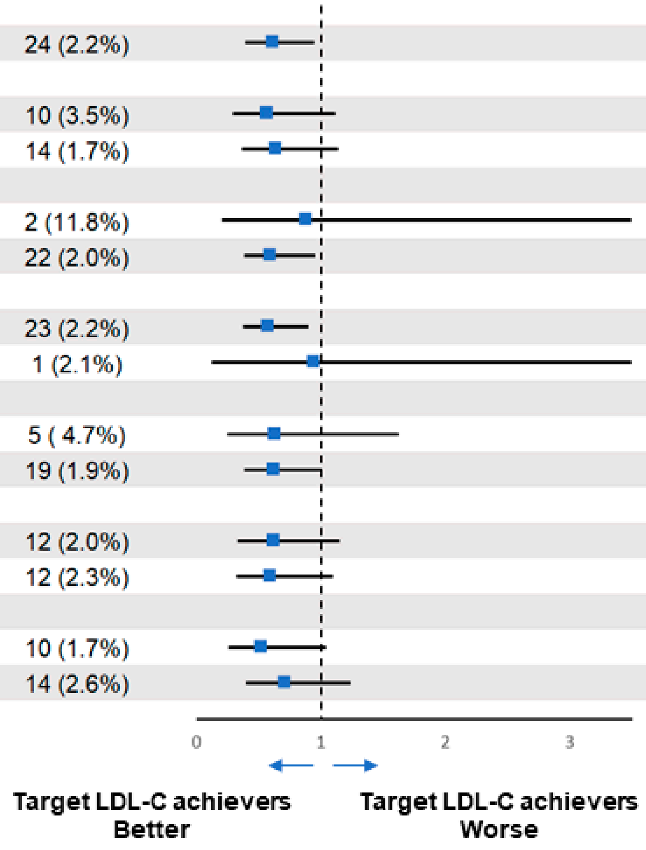

Supplement: Supplementary file 1 [file jcm-11-02650-s001.zip › jcm-1706616-supplementary.pdf]
